# Highly multiplexed, fast and accurate nanopore sequencing for verification of synthetic DNA constructs and sequence libraries

**DOI:** 10.1093/synbio/ysz025

**Published:** 2019-10-29

**Authors:** Andrew Currin, Neil Swainston, Mark S Dunstan, Adrian J Jervis, Paul Mulherin, Christopher J Robinson, Sandra Taylor, Pablo Carbonell, Katherine A Hollywood, Cunyu Yan, Eriko Takano, Nigel S Scrutton, Rainer Breitling

**Affiliations:** 1 Manchester Centre for Synthetic Biology of Fine and Speciality Chemicals (SYNBIOCHEM), Manchester Institute of Biotechnology, The University of Manchester, Manchester M1 7DN, UK; 2 School of Natural Sciences, Department of Chemistry, Faculty of Science and Engineering, The University of Manchester, Manchester M13 9PL, UK; 3 Institute of Integrative Biology, University of Liverpool, Liverpool L69 7ZB, UK

**Keywords:** synthetic biology, next-generation sequencing, DNA assembly, nanopore sequencing, strand bias

## Abstract

Synthetic biology utilizes the Design–Build–Test–Learn pipeline for the engineering of biological systems. Typically, this requires the construction of specifically designed, large and complex DNA assemblies. The availability of cheap DNA synthesis and automation enables high-throughput assembly approaches, which generates a heavy demand for DNA sequencing to verify correctly assembled constructs. Next-generation sequencing is ideally positioned to perform this task, however with expensive hardware costs and bespoke data analysis requirements few laboratories utilize this technology in-house. Here a workflow for highly multiplexed sequencing is presented, capable of fast and accurate sequence verification of DNA assemblies using nanopore technology. A novel sample barcoding system using polymerase chain reaction is introduced, and sequencing data are analyzed through a bespoke analysis algorithm. Crucially, this algorithm overcomes the problem of high-error rate nanopore data (which typically prevents identification of single nucleotide variants) through statistical analysis of strand bias, permitting accurate sequence analysis with single-base resolution. As an example, 576 constructs (6 × 96 well plates) were processed in a single workflow in 72 h (from *Escherichia coli* colonies to analyzed data). Given our procedure’s low hardware costs and highly multiplexed capability, this provides cost-effective access to powerful DNA sequencing for any laboratory, with applications beyond synthetic biology including directed evolution, single nucleotide polymorphism analysis and gene synthesis.

## Introduction

Synthetic biology is the engineering of biological systems for a desired outcome. These outcomes can include a variety of applications, including the production of therapeutics (1), fine chemicals (2, 3), biofuels (4, 5) and biomaterials (6, 7) or the generation of novel functional organisms like biosensors (8) or computers (9). Typically, synthetic biology requires the construction of synthesized DNA into specifically designed constructs, which are often large and complex. The discipline is fueled by the availability of inexpensive DNA synthesis and assembly methods (optionally involving high-throughput automation), allowing any designed sequence to be synthesized and assembled in a matter of days.

There are a wide variety of DNA assembly techniques available to create synthetic DNA constructs, including Ligase Cycling Reaction (LCR), Golden Gate, BioBricks, Gibson assembly (and other similar methods based on joining homologous ends) and recombination (10–12). For all of these methods, transformation of a host (e.g. *Escherichia* *coli*) permits the screening of clones to identify those harboring the correct assembly. Following this, identified constructs must be analyzed to verify that their sequence is identical to the intended design prior to testing for functional performance. Ideally, the sequencing of these large constructs is performed in-house, to allow tight coupling to the construct assembly and phenotyping platforms. Next-generation sequencing (NGS) is ideally suited to this task, given its ability to rapidly analyze gigabases of sequence data (13, 14). To date, NGS has been utilized in synthetic biology for a range of applications, including the design and analysis of synthetic biology parts (15–17), the characterization of genetic parts and circuits (18) and the study of DNA logic functions and circuits (19–21).

A number of NGS technologies are available, capable of generating both short- (notably Illumina, Ion Torrent) and long-read data (Pacific Biosciences and Oxford Nanopore) (22, 23). Sequence data derived from nanopore technology generates the longest read lengths currently available (often over 100 kb), allowing for the easy identification of repeating or moveable elements, which is challenging for short-read data (24). This would perfectly meet the needs of synthetic biology, which due to design-of-experiment (DoE) and combinatorial assembly approaches constructs can contain a large number of repeated elements and sequences (25, 26). However, the high-error rate of nanopore data (currently at 5–15%, compared to <1% for short read technologies) means that it is currently unsuitable for accurate detection of single nucleotide variants (SNV) and indels (27), unless combined with accurate short-read data (termed ‘hybrid assemblies’ (28–32)). Unfortunately, the large costs associated with installing high-accuracy sequencing technologies (e.g. Illumina or PacBio) limits the in-house accessibility of rapid and accurate NGS for small- to medium-sized synthetic biology laboratories.

This work describes a standardized workflow for the accurate sequence verification of synthetic DNA assemblies using nanopore technology, that is both quick and low cost and offers a solution to the problems created by the inherent high-error rate. The process involves polymerase chain reaction (PCR) amplification of assemblies directly from *E. coli* transformants to quickly obtain purified amplicons, nanopore sequencing to generate real-time data and a novel analysis algorithm to validate correct assemblies with high accuracy. Specific DNA barcodes are designed for the workflow, enabling highly multiplexed sequencing of hundreds of large constructs. Importantly, this novel analysis can accurately discriminate between systematic sequencing errors (inherent in high-error rate nanopore data) and genuine assembly mutations, permitting accurate SNV identification from high-error rate nanopore data. The process is designed to sequence any construct made using BglBrick vectors (33), a standardized set of *E. coli* expression plasmids widely used for the assembly of constructs in synthetic biology. To our knowledge, this is the first NGS workflow specifically designed to meet the needs of high throughput, multi-fragment DNA assembly approaches.

## Materials and methods

### Design of multiplexing primers

Universal primer binding sequences that are common to all the BglBrick vectors were first identified, such that they were upstream and downstream from the multiple cloning sites (MCS) of every construct (Supplementary Materials S1 and S2). Consequently, the same sequences could be employed to amplify any insert cloned into any of the BglBrick vectors. Whilst a terminal 3′ G was not required for all vector templates; it was found that its inclusion in the primer sequence significantly improved primer performance and it was therefore included in all designs.

For barcode design, 500 random 24-nucleotide sequences were designed and added to the forward and reverse universal primer binding site sequences to create the complete primer sequences. Primers were ranked according to their propensity to form secondary structure (highest Δ*G* ranked first), according to DINAMelt calculations (34). From the best-ranked sequences, 96 were selected as reverse barcode primers. 6 × 96 primer pairs were then created, providing 576 unique primer combinations, which can all be combined together for sequencing on one NGS run (see Supplementary Files). Further primers have been identified so that more than 6 forward barcode primers can be used to extend the multiplexing capability (Supplementary Material S3).

### Construct assembly

Specifically designed constructs were assembled using the LCR methodology as previously described (3, 35, 36). Transformant *E. coli* colonies were selected by automated colony picking into 1 ml Lysogeny Broth (with appropriate antibiotic added), covered with a breathable seal (Greiner) and incubated overnight at 30°C (950 rpm). Cultures were then diluted 1:400 in dilute phosphate buffered saline solution (13.7 mM NaCl, 1 mM sodium phosphate, 0.27 mM KCl, pH 7.4) to generate the PCR templates.

### Culture PCR for sample barcoding

PCR conditions were optimized for robust amplification from diluted overnight cultures. The best performance was obtained using CloneAmp HiFi (Takara Clonetech) high-fidelity polymerase, prepared as a 2× premix. Reactions contained 5 µl enzyme premix, 2.5 µl primer mix (containing 2 µM of both forward and reverse primers) and 2.5 µl diluted PCR template. PCR was performed using a 96 well thermocycler (Eppendorf Mastercycler) with an initial denaturation incubation at 95°C for 180 s, followed by 35 cycles of 98°C for 20 s, 64°C for 15 s and 72°C for 210 s, concluding with a final incubation of 72°C for 210 s. Amplicons were then analyzed by capillary electrophoresis, using the Fragment Analyzer Automated CE System and the dsDNA 930 reagent kit, following the manufacturer’s instructions.

### Sequencing

Amplicons selected for sequencing (entire plates or selected samples) were pooled and purified using the NucleoSpin Gel and PCR clean-up kit (Macherey Nagel). A total of 1–1.5 µg DNA was then prepared using the 1D amplicon/cDNA by ligation kit (SQK-LSK109, Oxford Nanopore) and sequenced using the MinION device (R9.4.1 flow cell) following the manufacturer’s instructions. During the MinION run the generation of real-time data permits rapid processing and analysis of data for the fast identification of correct assemblies (37). Typically, data were collected for up to 24 h and basecalling of the raw data was performed using Guppy (v2.3.7). More data could be obtained by increasing sequencing time to 48 h.

### Data analysis

A bespoke data analysis algorithm was constructed using both new and existing tools. The process is split into a number of steps: sequence reading, demultiplexing, alignment and analysis. Processed (basecalled) sequence data are initially read using Biopython (38). Demultiplexing is performed by custom code that is tolerant of the high-error rate typically found in nanopore data. Once demultiplexed, sequences are aligned to either their known target sequence (in the case of sequence verification) or all supplied target sequences (in the case of sequence identification) using the freely available Burrows-Wheeler Aligner program, BWA-MEM (39). Variant Call Format (vcf) files are then generated using SAMtools mpileup method (40), and the resulting vcf files are summarized to provide a measure of sequence identity, mutations, indels and maximum read depth for each of the individual samples within the pool.

The data analysis algorithm considers forward and reverse reads separately, due to the high frequency of strand bias in miscalling of bases. A Bayesian statistical strategy is introduced to ensure that SNVs are reported only if they appear concordantly on both strands. At positions of high-strand bias, indicating unreliable sequencing data, it is assumed that the nucleotide is most likely that of the template sequence. In the case of low strand bias and high data reliability, nucleotide calling probabilities are calculated, estimating the likelihood associated with each nucleotide for each read direction separately, using a binomial distribution, resulting in an implicit weighting by the number of reads on each strand. As a consequence, SNVs are only confidently reported with high probabilities if they are concordant on both strands and supported by an adequate number of reads. Source code and instructions are openly available at https://github.com/neilswainston/sbc-ngs/.

## Results and discussion

### Design of barcoded primers

The objective of this study was to develop an NGS workflow that could quickly and accurately determine host transformants exhibiting the correctly assembled synthetic DNA sequence. This process depends on PCR amplification of assemblies directly from the culture in order to quickly obtain concentrated DNA amplicons for sequencing, thus eliminating more laborious plasmid extraction protocols.

In order to sequence many discrete samples in a single experiment (multiplexing), NGS employs an indexing system based on DNA ‘barcodes’. Typically, barcode sequences are encoded at the termini of linear DNA either through ligation or PCR protocols. Following sequencing of pooled samples, each sample can then be demultiplexed, and assigned to their original sample based on these barcode sequences. Several highly multiplexed NGS systems have been developed, permitting the sequencing of hundreds to thousands of samples simultaneously (41). Notably, use of pairwise or asymmetric barcodes, where each terminus receives a specific barcode sequence, is a powerful way of economically processing many samples (42, 43). PCR is often utilized to create these specific combinations, given that primer pairs (encoding barcode sequences) can be combined prior to highly parallel PCR amplification of the target sequences. These primer sequences (encoding both the barcode and template-annealing sequence) must be empirically designed, such that they perform reliably and efficiently with their desired DNA template during the PCR. Consequently, there is a need for standardized workflows with specifically designed barcodes for amplicon sequencing, particularly for the inducible protein expression constructs typically used in synthetic biology.

**Figure 1.**
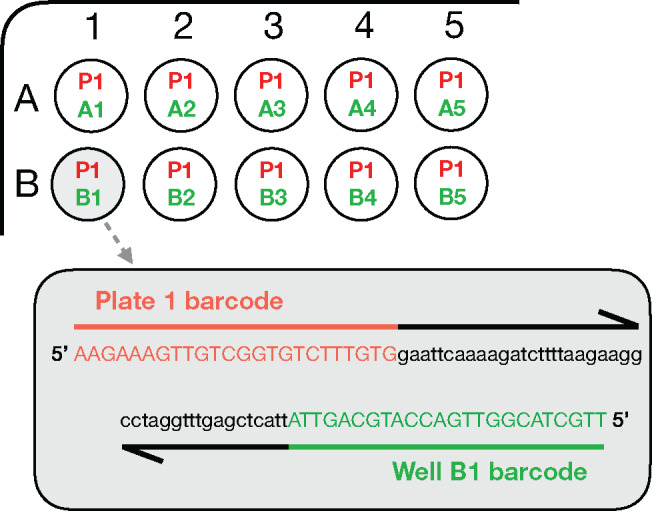
Allocation of primer pairs to enable the identification of individual wells from highly multiplexed samples, using well B1 from plate 1 as an example. Each well is allocated a forward primer, identifying the source plate, and a reverse primer, identifying the well. This enables the accurate identification of each individual well by data analysis after sequencing.

In this study, new barcoded primers for highly multiplexed nanopore amplicon sequencing were designed for use with the standardized BglBrick expression vectors. DNA assembly in synthetic biology often employs standardized BglBrick expression vectors, each containing a universal MCS (3, 33, 36). Therefore, generic primer binding sequences common to all the BglBrick vectors were selected, such that they were upstream and downstream from the MCS. Consequently, the same sequences could be employed to amplify any insert in any of the BglBrick vectors (Supplementary Material S1). A pairwise (asymmetric) multiplexing approach was employed, in which different 5′ and 3′ barcodes (encoded by the forward and reverse primers, respectively) identify the original sample location, whereby the 5′ barcode identifies the plate origin (termed ‘plate barcode’ primer) and the 3′ barcode identifies the well in that plate (termed ‘well barcode’ primer, Figure 1). Of all, 576 bespoke designed primer pairs (a total of six 96-well plates) were generated (see Supplementary Files); a number which can be increased with the introduction of further forward primers (Supplementary Material S3).

### PCR amplicon preparation and sequencing

Applying PCR directly from culture is the optimal method to isolate the barcoded construct sequences from a bacterial host, given that the alternative protocol of plasmid extraction, digestion/fragmentation and then barcoding is significantly slower, more labor intensive and costly. To this end, host transformant colonies were selected and cultured overnight, then prepared as PCR templates before addition to PCR reaction mixtures.

The high-fidelity polymerase CloneAmp HiFi (Takara Clonetech) was optimized to establish robust PCR conditions for amplifying constructs up to 10 kb. Every primer pair was tested using an exemplar 6.6 kb construct (Supplementary File) and provided efficient amplification of the target sequence, yielding a single PCR product of the correct size with high yield (Supplementary Material S4). Additionally, this amplicon length analysis provided an early screen for correct assemblies before sequencing, given that amplicons could be compared to their expected pathway lengths.

**Figure 2.**
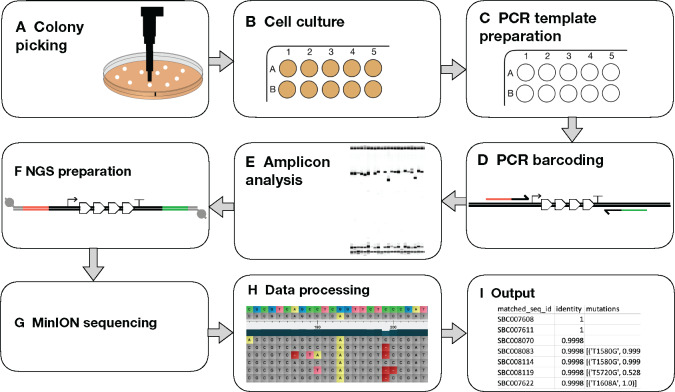
Overview of the construct-sequencing workflow. Colonies harbouring assembled plasmids are first (**A**) picked and (**B**) cultured in deep well plates, prior to (**C**) dilution to create the PCR template. (**D**) PCR amplification of the construct generates 5′ (red) and 3′ (green) barcoded amplicons which are (**E**) analyzed by capillary electrophoresis. (**F**) Pooled amplicons are prepared for NGS sequencing by adapter ligation and (**G**) sequenced using the MinION device. (**H**) Bioinformatics processing of data identifies mutations and removes systematic errors by probabilistic analysis and (**I**) data metrics are outputted.

Having obtained barcoded amplicons, these are pooled and purified, then prepared for nanopore sequencing. This procedure involves end preparation, adapter ligation and incubation with the motor protein, a process that prepares the DNA for translocation through the nanopore during the sequencing run (Figure 2).

### The data processing algorithm for accurate sequencing using nanopore data

A major limitation of nanopore sequencing data is its low single-passage strand sequencing accuracy, manifesting in a high-error frequency (44, 45). Simple alignment of these erroneous ‘noisy’ sequences generates a consensus sequence, which given sufficient read depth is capable of eliminating almost all of the sequencing error. However, systematic errors in the data are difficult to remove from standard consensus alignments (46), restricting these data for use in accurate SNV detection. Indeed, in this study some constructs known to be correct (tested by Sanger sequencing) were identified as having only 99.5–99.9% identity to the target sequence from consensus alignment of all sequencing reads, with one or more systematic sequencing errors preventing complete identity.

It is believed that systematic errors can arise from either specific sequences with indistinguishable conductance signals or motor enzyme processing errors (45). As both these events could be sequence-specific, we hypothesized that the occurrence of these errors could vary between the strands being passaged, given that the lower strand is passaged as the reverse complement sequence of the upper strand. For example, if a miscalling occurs at the end of a hairpin in a top strand read, the bottom strand read would correctly basecall this sequence before the hairpin is encountered. This type of strand bias is known to occur for various sequencing platforms including Illumina and Nanopore (47, 48); however, to date it has only been exploited for improving SNV accuracy by McElroy *et al*. (49) using data derived from Roche-454 technology.

**Figure 3.**
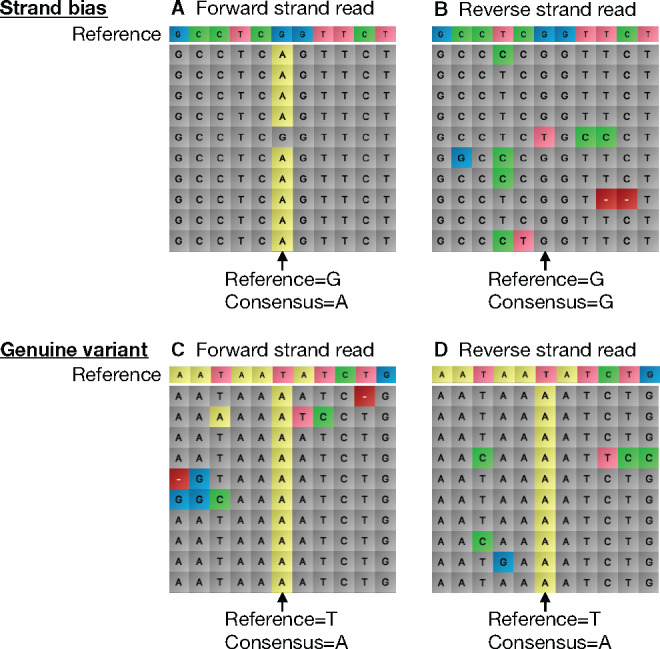
Examples of strand bias and a genuine SNV in nanopore data. Each row corresponds to a different read. Bases correctly aligned to the target sequence are shown in gray and potential mutations are highlighted in color (A = yellow, G = blue, T = pink, C = green, deletion = red). The consensus basecall is identified at the relevant position. Strand bias is shown by the inconsistent SNV basecalling between the (**A**) forward and (**B**) reverse strand reads from the same sample. Our statistical analysis of this alignment prevents erroneous SNV identification. In contrast, genuine SNVs are identified by an agreement between the (**C**) forward and (**D**) reverse strand read data.

Strand bias in nanopore data can be identified through separate alignment of forward and reverse strand reads (Figure 3). Where bias occurs, an SNV is identified in one strand with high frequency whilst the corresponding strand read identifies the non-variant base as the most frequent. This strand bias is exploited in our Bayesian statistical analysis to accurately distinguish between true SNVs and systematic sequencing errors through a probabilistic comparison of forward and reverse strand reads (see Materials and methods section). This therefore enables the analysis to identify when an SNV identified is true (i.e. occurs in both top and bottom strand reads) or miscalled (occurs in one but not both reads). To our knowledge, this is the first example of analyzing and exploiting strand bias in a nanopore analysis workflow for accurate SNV identification.

### Experimental verification of the multiplexed workflow

To verify the robustness of our workflow, each of the 576 primer pairs was validated using a control construct (a pinocembrin-producing metabolic pathway (3)). Under experimental conditions, each of the 576 primer pairs demonstrated robust amplification of the target construct with high yield by long-range PCR using the diluted bacterial culture directly as a template (see above and Supplementary Material S4). Analysis showed that each of the 576 samples was identified with 100% coverage and no reported misreads or mutations (identity = 1). It was encouraging to observe that the lowest read depth in this dataset was 46 (total of both forward and reverse strands) yet was still reported as correct, demonstrating that this workflow can accurately identify correct assemblies from a low number of consensus reads (Supplementary Materials S5 and S8).

Demonstration of our workflow for experimental sample sequencing was performed on a library of 563 assembled constructs. Analysis of PCR amplifications by capillary electrophoresis (Supplementary Material S6) showed variation in amplicon size, indicating that some constructs were reliably assembled by LCR, whilst others were not correctly assembled (one or more DNA parts missing). This inconsistency is caused by LCR assembly efficiency, which can be significantly hampered by the number, size, sequence and complexity of the DNA parts being assembled. Upon sequencing, a total of 128 from 563 test assemblies were verified as fully correct sequences with 100% nucleotide identity (Supplementary Materials S7 and S8), in agreement with the size approximations from the electrophoresis analysis (Supplementary Material S6).

## Concluding remarks

In this study, a standardized protocol for highly multiplexed DNA sequencing of assembled constructs for synthetic biology is presented. Using PCR amplification directly from bacterial cultures, barcoded amplicons are generated that are ready for sequencing, enabling the rapid processing of hundreds of samples in parallel. Currently, the process is capable of processing data for 576 samples in 72 h (this can be shorter when preparing fewer samples). This process is therefore not only quicker and higher throughput than conventional Sanger sequencing, but cost per sample is also significantly lower. In the workflow, the total cost per sample for the entire workflow is £2.20 ($2.73) regardless of construct length. For the control experiment (6.6 kb amplicon per sample), the price per kb was £0.33 ($0.41), which is substantially cheaper than the equivalent length Sanger sequencing (currently £7.77 ($9.64) per kb, Supplementary Material S9). Taken together, with the hardware acquisition of the nanopore (the device is provided as part of a ‘starter pack’), this presents an attractive low-cost means to perform NGS in-house for small- to medium-sized laboratories.

In addition to cost, this workflow provides other novel features. First, an optimized set of barcoding primers are provided, designed such that they can be repeatedly used with any of the 96 expression plasmids from the BglBrick library (33). While our example study demonstrates the workflow using this library, the same primer design approach is equally applicable to other sets of expression plasmids (by transferring the barcode sequences to any plasmid-specific primer sequence). Furthermore, the design is amenable to processing more samples by adding additional primers sets (further sets for up to 12 × 96 well plates are described in Supplementary Material S3). Aside from the pathway sequencing shown here, this workflow can also be easily applied to other sequencing applications, such as variant libraries in directed evolution and single nucleotide polymorphism analysis and identification of correct sequences during gene synthesis.

Additionally, a powerful informatics algorithm is provided for automated processing of FASTQ files. Typically, a lack of relevant bioinformatics expertise to process NGS data often prevents laboratories from utilizing this powerful technology in-house, and therefore this algorithm provides a useful means for them to exploit this technology. In the analysis of the control dataset, it was found that a read depth of as little as 46 reads was sufficient for accurate sequence verification. Given that the total number of reads (with both barcodes identified) obtained in 24 h were >1 329 000, a theoretical sequencing capacity is calculated of over 10 000 constructs. However, in order to achieve this throughput further effort is required to normalize the sample concentrations to ensure sufficient reads are obtained for every barcode set. Given the continuing demand for higher throughput DNA assembly capability in synthetic biology, the approach introduced here provides the community with a powerful resource for fast multiplexed DNA sequencing and analysis at a dramatically lower cost than for other sequencing technologies.

## Availability

All NGS data and processed results described in this work are freely available at https://console.cloud.google.com/storage/browser/sbc-ngs/. Source code, the SBC003382 plasmid sequence and instructions for use are openly available at https://github.com/neilswainston/sbc-ngs/.

## Author contributions

A.C., N.S., M.D., A.J.J., C.J.R., S.T., P.C., K.A.H. and C.Y. conceived the project, experimental design and performed experimental work. N.S. and R.B. wrote the analysis algorithm. P.M. established the data management. A.C., N.S., E.T., N.S.S. and R.B. compiled and edited the manuscript and supervised the project.

## Supplementary Material

ysz025_Supplementary_Data
