# Supplementary material for: Highly multiplexed, fast and accurate nanopore sequencing for verification of synthetic DNA constructs and sequence libraries
Source: Synth Biol (Oxf). 2019 Oct 29;4(1):ysz025. doi: 10.1093/synbio/ysz025 (PMC7445882; doi:10.1093/synbio/ysz025)
Supplement: ysz025_Supplementary_Data [file ysz025_supplementary_data.zip › Supplementary Material S1-S3.docx]

# S1. Sequence map of SBC003382 plasmid used for control samples. Amplification using the generic forward and reverse primer sequences produces an amplicon of 6580 bp.

# S2. Generic primer sequences added to the 3’ of each forward and reverse primer. The base shown in brackets was included in the sequence despite not being required for every plasmid in the BglBrick set. However, primer performance was found to be superior when this was included, compared to primers with this base omitted.

| Primer direction | Sequence |
| --- | --- |
| Forward | GAATTCAAAAGATCTTTTAAGAAG(G) |
| Reverse | TTACTCGAGTTTGGATCC |

# S3. Barcode sequences

# Plate barcode sequences used in this study, barcodes 1 to 6 were used in this study but 7-12 are shown for further multiplexing.

| Plate | Sequence |
| --- | --- |
| PL01 | AAGAAAGTTGTCGGTGTCTTTGTG |
| PL02 | TCGATTCCGTTTGTAGTCGTCTGT |
| PL03 | GAGTCTTGTGTCCCAGTTACCAGG |
| PL04 | TTCGGATTCTATCGTGTTTCCCTA |
| PL05 | CTTGTCCAGGGTTTGTGTAACCTT |
| PL06 | TTCTCGCAAAGGCAGAAAGTAGTC |
| PL07 | GTGTTACCGTGGGAATGAATCCTT |
| PL08 | TTCAGGGAACAAACCAAGTTACGT |
| PL09 | AACTAGGCACAGCGAGTCTTGGTT |
| PL010 | AAGCGTTGAAACCTTTGTCCTCTC |
| PL011 | GTTTCATCTATCGGAGGGAATGGA |
| PL012 | CAGGTAGAAAGAAGCAGAATCGGA |

**Well barcode sequences used in this study**

| WellPosition | Sequence |
| --- | --- |
| A1 | TGTGTTGAGACCACACAGGCCTCA |
| B1 | GTCTGTCGCCATGGAAAGTCAACT |
| C1 | TTGCTACGGTTGACCATGCAGTTA |
| D1 | AACTTGAGGTATCGTATATTCAAT |
| E1 | GCAGGTGGGCATCCGGACCGATAT |
| F1 | CAGAGCTGACCCTCCAGATATTTG |
| G1 | TCTTAGTGTATGAGCTCGCTCACC |
| H1 | CCCTGGGACGTAGGAATCCACGCC |
| A2 | TGTTGCGAACGGGACCTGCCTAGC |
| B2 | ACACCTTTACATAGGCCGCCATCT |
| C2 | GACCTTAGTCACATGGTAGTCTAA |
| D2 | GTTCGGATGCAATATGGTTCACTG |
| E2 | TAGCAGAAGTCCCTGTAAGACCAT |
| F2 | GATTCTGATTACTCTATTCGCCAG |
| G2 | GGAATAATACCATTGAAGTAGCAC |
| H2 | GGGTCCCTCTACTCATTTAGCATG |
| A3 | ATAGCTGAAGCAATCTACCTATCG |
| B3 | AGCACCTTCTTCGGATCAGTTGTT |
| C3 | CGGGCTCATTGCGATTTCTATGCC |
| D3 | CGGGCCCTTCATCTCTCAGCCGAT |
| E3 | CAGAGTAAGGGTATAGGTTCGGCA |
| F3 | CGACTACCCAACGACGCAGTAATG |
| G3 | GGCGGGTTCCTGTCGTTGTTAACT |
| H3 | CAATCAACGAATTAGATGTCGGGT |
| A4 | CGAATGTGCTGAGATTAGCTTGCA |
| B4 | TAGGGTTTCCGAAGTCAGCGTTCA |
| C4 | TTGACTAGTGCGTTTATTGGACGA |
| D4 | TTGTAGAATGGGAGACTCACCGAT |
| E4 | ATTGCATAGGAGGGTGCTTCGCAA |
| F4 | GCATGATTCCTGCTATTTGTCAGC |
| G4 | ATACATGGCTAATCTTGAAGCAGG |
| H4 | CTCCCGTAGGCATTGACCTGTTCA |
| A5 | TTGACGCGCTACTAATACGTTCTG |
| B5 | AGCGACATAATGGTATCAAATACC |
| C5 | TGACTATTTGATTAAGATAATCGG |
| D5 | GAACGTCGAAACCGCGATGCCGTT |
| E5 | TTGTCAGGCTCAGTCACCCTTCCG |
| F5 | GCATTCTTGTGTGTACGTCAATGC |
| G5 | GGAAGTCCGATCCGTATTTGCCTA |
| H5 | CTTAGGCGGATAATGTTGGCGAAA |
| A6 | CGGATACTAAGTTATCCTGCTGCG |
| B6 | TCATCGGTTAACGTACATCTGCGC |
| C6 | ACTCGCTTTCTAAAGTTGACACAC |
| D6 | GGCTCATATGTAACAAGCAGTAGG |
| E6 | TCATTTCCTAAGACTTACAGAGGT |
| F6 | TCGTTTGCTACACAATGTCGTTGC |
| G6 | GGGATCTAGATACCTCTTGCGTGT |
| H6 | TAGGTTCAACGCGTCTGCCACCGT |
| A7 | ACTTGCTTGTCATTGAAACGCTAC |
| B7 | GAGAGCGTCTATTCTCAGTTAATG |
| C7 | ATCGTTGAGTTGATTAGCACACGG |
| D7 | CTCCTGAATACATGGAGCTCCAAC |
| E7 | CGCATGAACCGGAACTATAGGCGT |
| F7 | GGAGGTGACTGCATTAAAGTGAGT |
| G7 | GGATTGACCGGGATGGCATGAGGA |
| H7 | GTCAACTCACCTCAGAGAATCTAC |
| A8 | CGGGACCTCCAGAAATCTAGTCGA |
| B8 | GGCTGGCCAGTTGCAGAAGCAGTC |
| C8 | TTGGGTATAAATGATGTGGTGACA |
| D8 | CCTATGCTTTGGACCCGACAAGGA |
| E8 | TTCTGTAGATATGGGTAAGTTTGC |
| F8 | TCTGGCATGAATCAGCAGAGATTG |
| G8 | CAGTGGCACTTTAGTCACTCTAAT |
| H8 | CAGATGTCCGAACTACTAACCTAC |
| A9 | AGGCTCTTGATCCAGAGACCGCGC |
| B9 | CCGGAGAAAGGATCAGTGGCCTAT |
| C9 | CGCTGGAACTTATCACTGAGTGTC |
| D9 | AGGAAACTCAATCTGGGTTGCACC |
| E9 | CGGTCTTAGTGCGATATATGCGGC |
| F9 | CTTAGTCAGCGCCATGGGACCATA |
| G9 | CAGAAAGAATGTACTAGCTCGGAG |
| H9 | GGATTTCACAACATAATGTTTGGA |
| A10 | GGTTCCTTTCTACTTATCCGTAAT |
| B10 | CAAGTCGGTGCAGGTACACTAGGC |
| C10 | TCGTTCCTGGATTTAGCTAACTAG |
| D10 | CTCGACCTCACTCCCAGTGGCGGC |
| E10 | CAGGCAAACGGGATCTCGTGGACA |
| F10 | TATAGTTTCAGCTCCTTATTGGCG |
| G10 | ATATATAGGGTGCCATGAGACGAA |
| H10 | TGCATACAGTTAAATTCATTGAGG |
| A11 | ATCCGGCCAGACAGTCAGGGACAT |
| B11 | AAGTTAGCGAGATATAGGCTCACC |
| C11 | GATGAGACAGACAACACACACGCA |
| D11 | GAGATGTGAGACAAGCATTCAAAG |
| E11 | ACACTGTGTTCCACCCGATGTGAC |
| F11 | TGGAGCTCGAATTGCCTATTCACC |
| G11 | AAGTGTACTAGGTGCAATCGGACC |
| H11 | CGCCTCATTCGGATTCTATGGCAG |
| A12 | GTTCGTGTAATCCCTACCCGTGTT |
| B12 | TACTAGCCACCTCCTCCCTCATCG |
| C12 | AAGTTCATAACTGGTAGCAATGTA |
| D12 | CATGTACAATGCAGGCGGATCACG |
| E12 | TATGTTACCTGTTCCGACGTAAGC |
| F12 | TAGTAATCGAGAGTGCCATCCTGT |
| G12 | ATTCTCGCGCGTCGGTGGTTGGTC |
| H12 | GTGGGAAGTGGCTTGTCACCTTCA |
