# Supplementary material for: Highly multiplexed, fast and accurate nanopore sequencing for verification of synthetic DNA constructs and sequence libraries
Source: Synth Biol (Oxf). 2019 Oct 29;4(1):ysz025. doi: 10.1093/synbio/ysz025 (PMC7445882; doi:10.1093/synbio/ysz025)
Supplement: ysz025_Supplementary_Data [file ysz025_supplementary_data.zip › Supplementary Material S4.docx]

# S4. Capillary electrophoresis analysis of the PCR amplification of control pathway (SBC003382).

Plate 1

Plate 2

Plate 3

Plate 4

Plate 5

Plate 6
