# Supplementary material for: Highly multiplexed, fast and accurate nanopore sequencing for verification of synthetic DNA constructs and sequence libraries
Source: Synth Biol (Oxf). 2019 Oct 29;4(1):ysz025. doi: 10.1093/synbio/ysz025 (PMC7445882; doi:10.1093/synbio/ysz025)

# S6. Capillary electrophoresis analysis of the PCR amplification of LCR assembly samples.

Plate 1


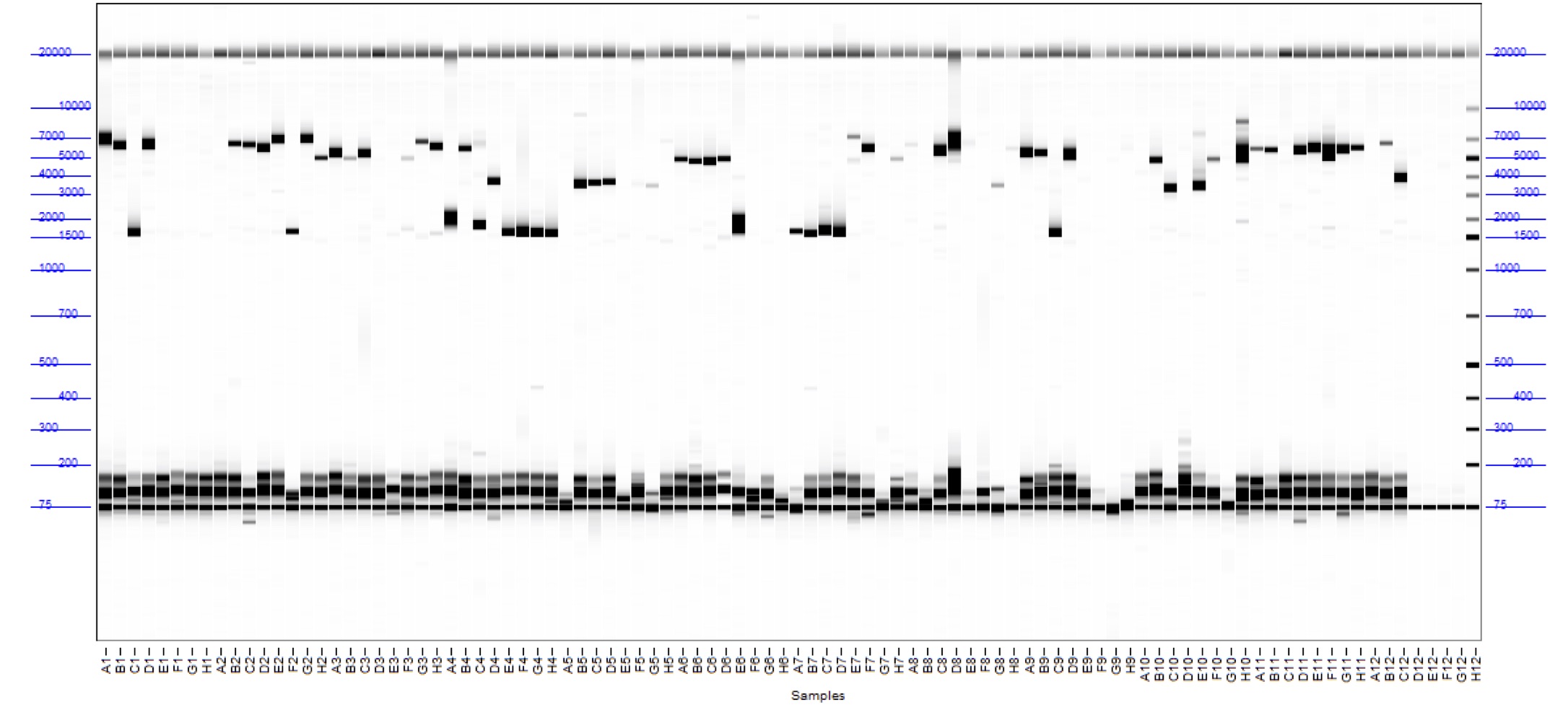


Plate 2


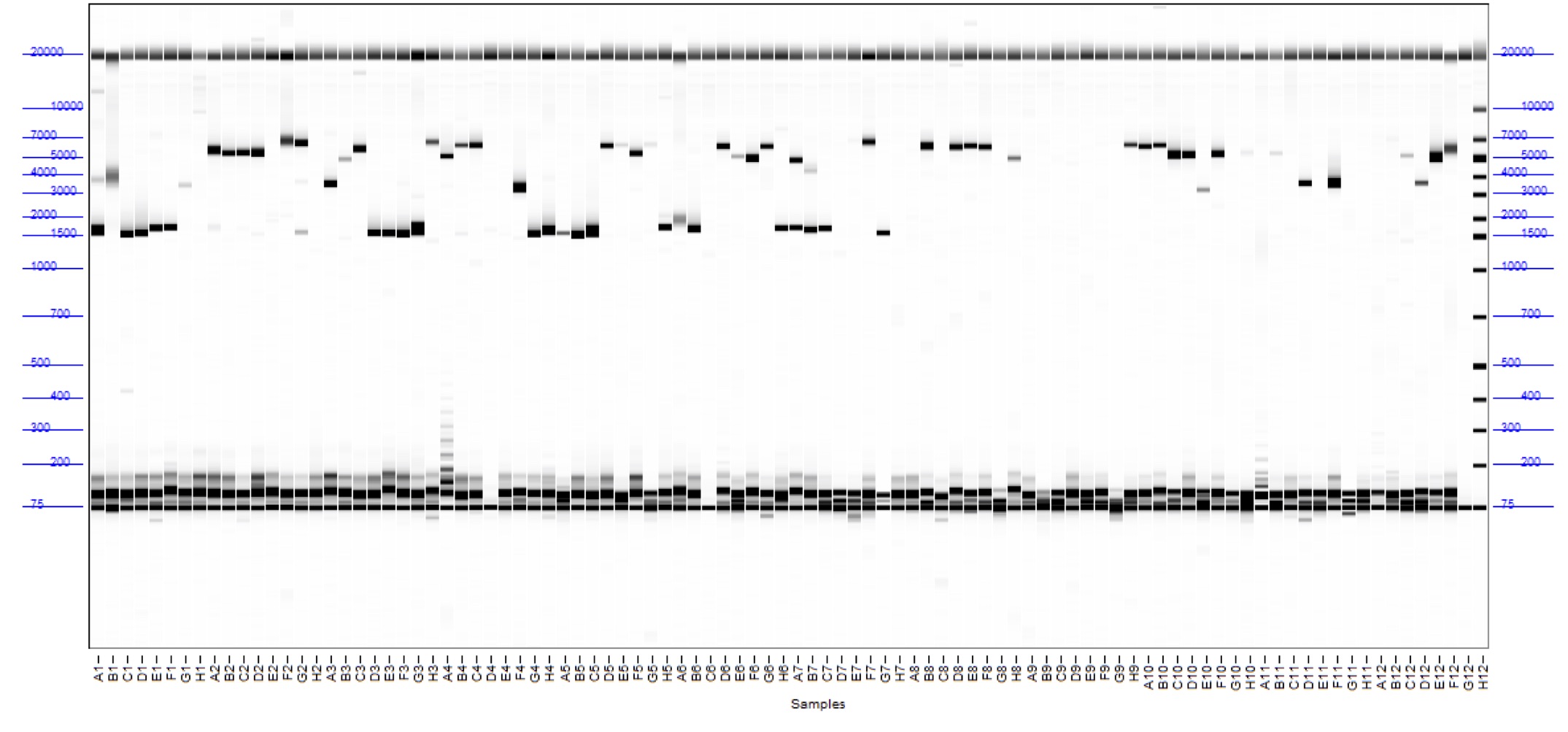


Plate 3


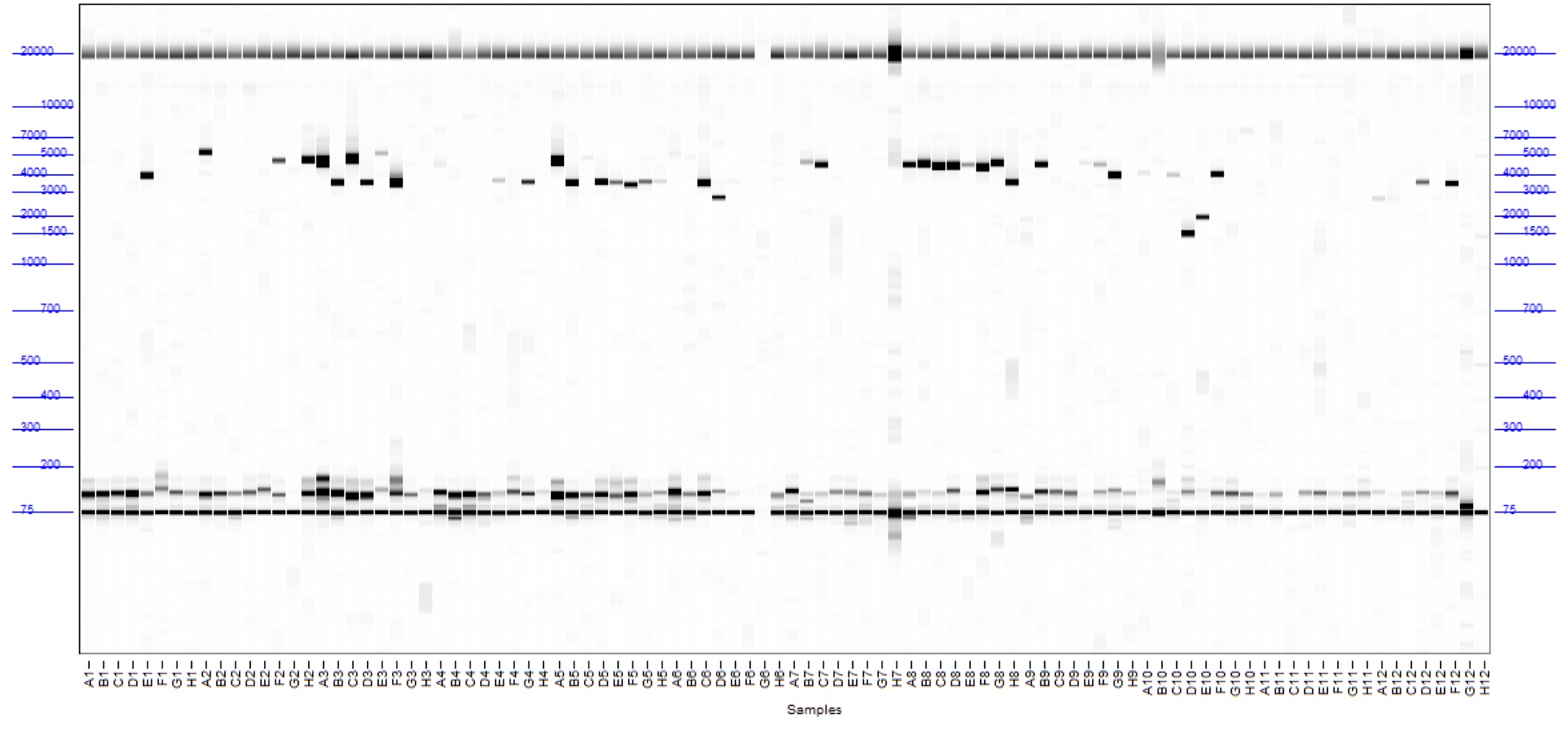


Plate 4


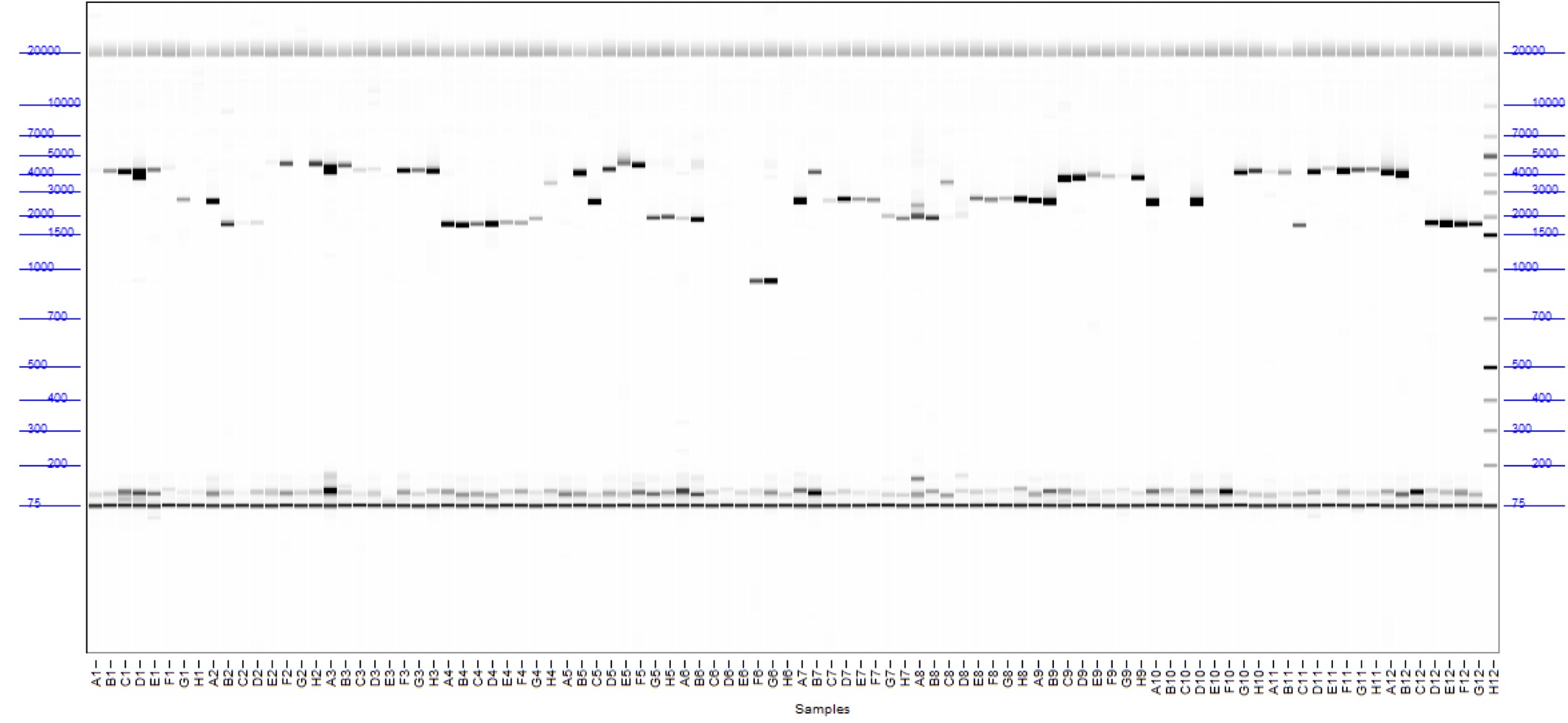


Plate 5


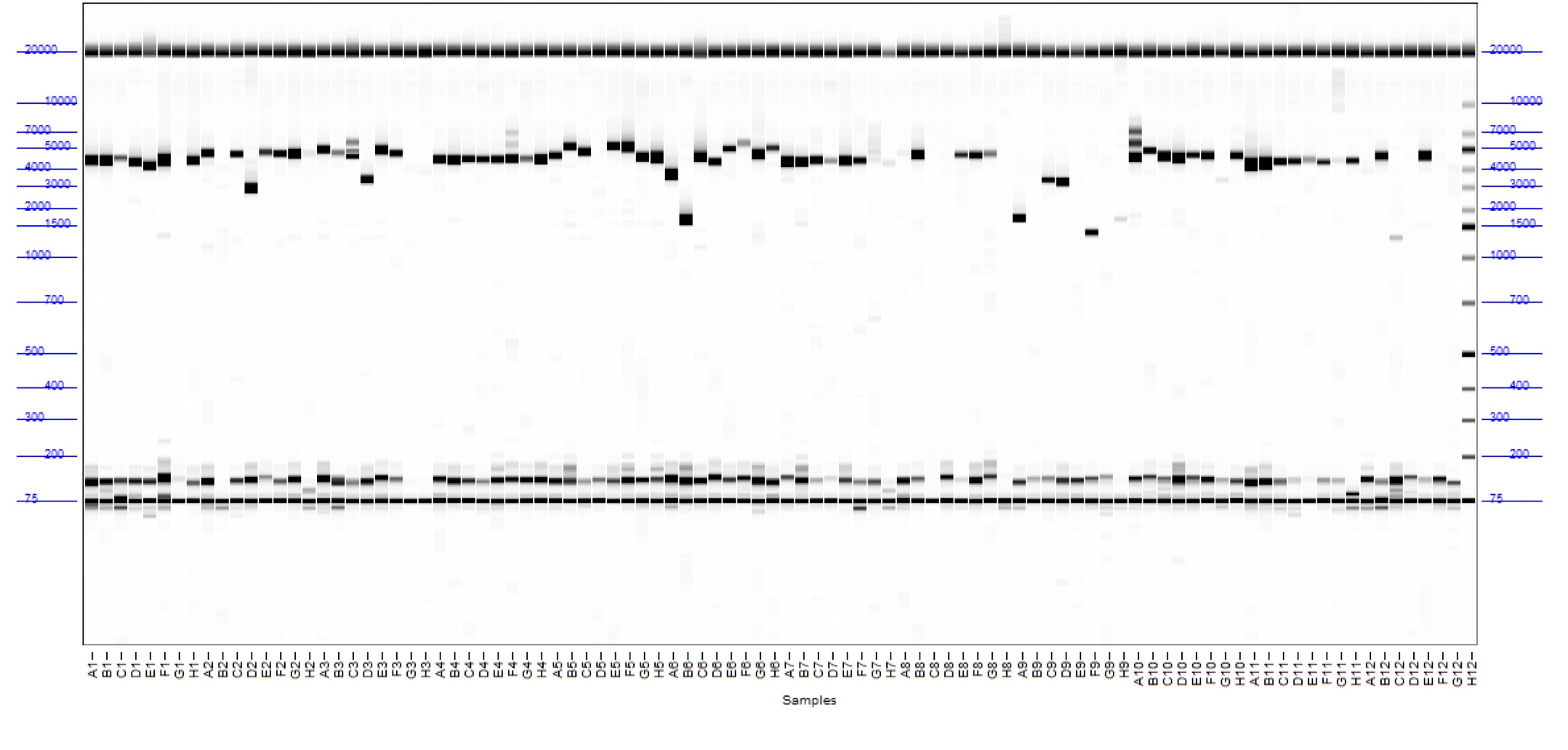


Plate 6


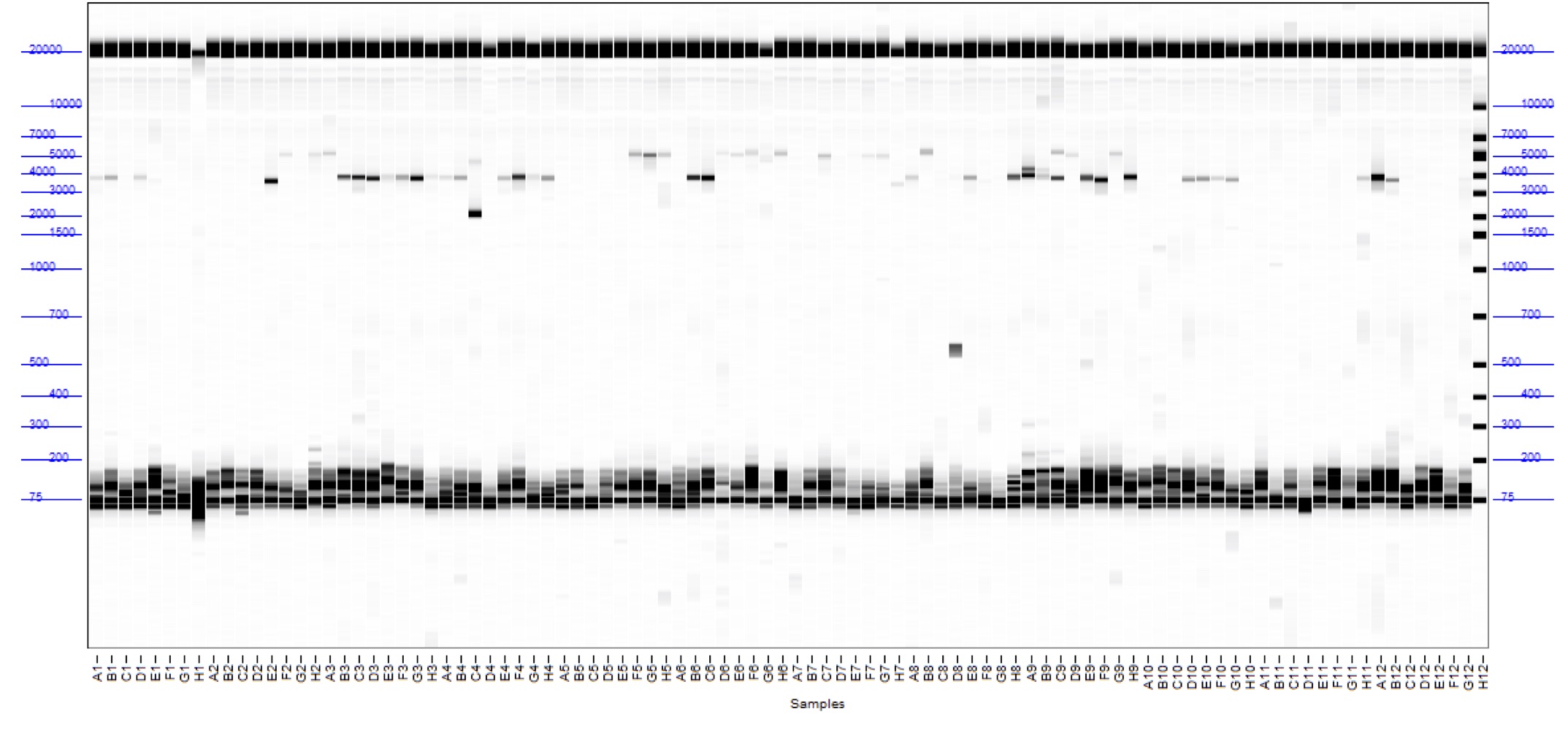

Supplement: ysz025_Supplementary_Data [file ysz025_supplementary_data.zip › Supplementary Material S6.docx]
