# Supplementary material for: Highly multiplexed, fast and accurate nanopore sequencing for verification of synthetic DNA constructs and sequence libraries
Source: Synth Biol (Oxf). 2019 Oct 29;4(1):ysz025. doi: 10.1093/synbio/ysz025 (PMC7445882; doi:10.1093/synbio/ysz025)
Supplement: ysz025_Supplementary_Data [file ysz025_supplementary_data.zip › Supplementary Material S8-S9.docx]

# S8: Results output format.

The following applies to results output, including those of Supplementary Material S5 and S7.

| Column | Description |
| --- | --- |
| forward | The forward strand barcode. |
| reverse | The reverse strand barcode. |
| well | The well number. |
| known_seq_id | The known sequence id. That is, the id of the sample at this well position if it is pre-known. (If not, the algorithm will identify the sample id from a supplied list of sample ids and FASTA files of the associated sequences.) |
| matched_seq_id | The matched sequence id. If a ‘known_seq_id’ is supplied, this value will be the same as the known_seq_id, and the algorithm will attempt to verify the sequence rather than identify it. If no ‘known_seq_id’ is given the algorithm will identify the sample if from a supplied list of sample ids and FASTA files of the associated sequences and report the sample id here. |
| identity | The proportion of bases correctly identified in comparison with the template sequence. (A value of 1.0 indicates a fully verified sequence.) |
| mutations | A list of pairs of suspected mutations and their likelihood score, ranging from 0 (low confidence) to 1 (high confidence). This is the score given by the strand-specific analysis method. |
| deletions | A list of ranges of deletions identified in the aligned sample sequences. |
| depths | The maximum read depth across the aligned sample. Provides an indication of how many reads contributed towards the alignment. |

**S9: Workflow cost**
