# Supplementary material for: Highly multiplexed, fast and accurate nanopore sequencing for verification of synthetic DNA constructs and sequence libraries
Source: Synth Biol (Oxf). 2019 Oct 29;4(1):ysz025. doi: 10.1093/synbio/ysz025 (PMC7445882; doi:10.1093/synbio/ysz025)
Supplement: ysz025_Supplementary_Data [file ysz025_supplementary_data.zip › DATA AVAILABILITY.docx]

**DATA AVAILABILITY**

<https://github.com/neilswainston/sbc-ngs/>
